# Role of Akirin in Skeletal Myogenesis

**DOI:** 10.3390/ijms14023817

**Published:** 2013-02-08

**Authors:** Xiaoling Chen, Zhiqing Huang, Huan Wang, Gang Jia, Guangmang Liu, Xiulan Guo, Renyong Tang, Dingbiao Long

**Affiliations:** 1 Key Laboratory for Animal Disease-Resistance Nutrition of China Ministry of Education, Institute of Animal Nutrition, Sichuan Agricultural University, Chengdu 611130, Sichuan, China; E-Mails: xlchen@sicau.edu.cn (X.C.); hwangsc@163.com (H.W.); gangjcn@126.com (G.J.); lguangmang@163.com (G.L.); 2 Sichuan Key Laboratory of Meat Processing, Chengdu University, Chengdu 610106, Sichuan, China; E-Mail: xlguotang@163.com; 3 Faculty of Bioindustry, Chengdu University, Chengdu 610106, Sichuan, China; E-Mail: tangryg@163.com; 4 Chongqing Academy of Animal Science, Chongqing 402460, China; E-Mail: dingbiaolong@126.com

**Keywords:** Akirin, skeletal myogenesis, tissue distribution, signal pathway

## Abstract

Akirin is a recently discovered nuclear factor that plays an important role in innate immune responses. Beyond its role in innate immune responses, Akirin has recently been shown to play an important role in skeletal myogenesis. In this article, we will briefly review the structure and tissue distribution of Akirin and discuss recent advances in our understanding of its role and signal pathway in skeletal myogenesis.

## 1. Introduction

Skeletal myogenesis is a multistep process in which multipotent precursor cells give rise to myoblasts that subsequently withdraw from the cell cycle, and differentiate and fuse into multinuclear myotubes and then myofibers [1,2]. Skeletal myogenesis is mainly regulated by the muscle-specific transcription factors, including MyoD, myogenin, myogenic factor 5 (Myf5), myogenic regulatory factor 4 (MRF4) and myocyte enhancer factor-2 (MEF2) [3,4]. Skeletal myogenesis is also controlled by various autocrine/paracrine growth factors and cytokines, such as myostatin [5]. However, the control of skeletal myogenesis is not only restricted to the above mentioned factors, but also might have some novel factors.

Akirin is a nuclear factor required for innate immune responses [6]. The *Akirin* gene is highly conserved among vertebrates and has two homologues, *Akirin1* and *Akirin2* [7]. Till now, the *Akirin* gene was cloned from different species such as mice, duck, chicken, turbot and pig [6,8–13] and its expression pattern has been previously examined in different species [8–11,14,15]. Beyond its role in innate immune responses, Akirin has recently been shown to play an important role in skeletal myogenesis [14–19] and be negatively regulated by myostatin in skeletal muscle [14]. In addition, analysis of single-nucleotide polymorphism (SNP) in Japanese black beef cattle reveals that *Akirin2* is regarded as a positional functional candidate for the gene responsible for marbling [20]. In this review, we will briefly introduce the finding, structure and tissue distribution of Akirin, the basic biological role of Akirin in skeletal myogenesis and its possible signaling pathway.

## 2. The Finding and Structure of Akirin

In 2008, *Akirin* was firstly isolated from *Drosophila melanogaster* using a genome-wide screen by RNA-mediated interference [6]. The *Akirin* gene is conserved in vertebrates and at least two homologues, named *Akirin1* and A*kirin2*, have been identified [6]. However, the *Akirin* gene family comprises a single gene (*Akirin2*) in birds/reptiles, and the teleost species have two to eight Akirin family members [6,7,15,17].

The Akirin protein contains 180–204 amino acid residues with a predicted molecular weight about 20–25 kDa [17]. Homology analysis revealed that the conservation region of Akirin protein exists at the putative N-terminal and C-terminal domains, and a less conservative sequence locates in the middle region of the protein [6,7,17]. All Akirin protein sequences contain a highly conserved N-terminal nuclear localization signal (NLS) (Pro-Val-Lys-Arg-Arg), a functional motif, and Akirin was strictly localized to the nucleus [6,7,17].

The *Akirin* sequence has no obvious DNA- or RNA-binding motifs, so it cannot directly bind DNA [6], but it can interact with cofactors to promote or repress mRNA transcription including 14-3-3 proteins [21]. The 14-3-3 proteins are phosphoserine/threonine-binding proteins. It has been reported that 14-3-3 proteins can regulate subcellular localization, protein-protein interactions of target proteins, and many cellular activities including the cell cycle, intracellular signaling, apoptosis and malignant transformation [21–23]. There is a growing body of evidence indicating that *Akirin2* physically interacts with 14-3-3 proteins in the nucleus to regulate gene expression [7,21]. However, *Akirin1* has several low affinity 14-3-3 binding sites [7] and was detected in the nucleus [6], suggesting that the positive function of the Akirin1 may be mediated through binding 14-3-3 proteins [7]. Therefore, an interesting line of future investigation will be to further explore the relationship between *Akirin* and 14-3-3 proteins and whether the role played by Akirin is accounted for by binding to 14-3-3 proteins.

## 3. The Tissue Distribution of *Akirin*

A large number of studies show that the *Akirin* is expressed in a variety of different tissues [8–11,14,15]. Marshall *et al.* found that mouse *Akirin1* was expressed in many tissues such as brain, testes, lung, kidney, intestine and liver [14]. They also found that *Akirin1* expression was comparatively low in skeletal muscle, while its expression was increased in myostatin-null skeletal muscle [14]. In addition, the *Akirin1* expression was detected in inflammatory cells macrophages from peritoneal cavity and bone marrow of mice [16], which imply that Akirin may play an important role in inflammation reaction. More recently, data from our group showed that porcine *Akirin2* mRNA was mainly expressed in the lung and modestly expressed in the skeletal muscle and heart [9]. Man *et al.* reported that chicken *Akirin2* mRNA was highly expressed in the brain and oviduct [8]. Moreover, the expression levels of eight *Akirin* family members were detected in different tissues of Atlantic salmon [15]. These findings suggest that the functions of the Akirin are presented diversely in different tissues and different developmental stages among different species.

## 4. Effects of Akirin on Skeletal Myogenesis

Several lines of evidence support a role of Akirin in skeletal myogenesis. First, the expression of Akirin is negatively regulated by myostatin [14,16]. Second, Akirin has been reported to promote myogenic differentiation [14,19]. Finally, Akirin has been shown to induce the quiescent satellite cells (SC) activation and migration [14,18].

### 4.1. Akirin Expression is Negatively Regulated by Myostatin

Myostatin, a member of transforming growth factor β (TGF-β) superfamily, is a negative regulator of skeletal myogenesis [3]. Myostatin is predominantly expressed in skeletal muscle tissue. Loss of myostatin function results in a widespread increase in skeletal muscle mass due to both muscle hypertrophy and hyperplasia [3,14,24–26].

After a suppression subtract hybridization (SSH) strategy was used to compare differentially gene expression between myostatin-null mouse muscle and wild-type mouse muscle, a novel gene named *Akirin1* was isolated and its expression was reportedly only increased in the skeletal muscle of myostatin-null mice [14,16]. Moreover, the *Akirin1* expression has been shown to be suppressed by myostatin through MEK/ERK signaling pathway [14]. However, the inhibition is partially rescued by the antagonistic effect Mstn-ant1 (myostatin cDNA was truncated at the amino acid 350, produing a truncated portion of the processed region of amino acids 266–350) on myostatin activity [14]. Taken together, these results suggest that Akirin1 is negatively regulated by myostatin and plays a key role in the signaling pathway of myostatin.

### 4.2. Akirin Increases the Expression of MRFs

The muscle specific bHLH transcription factors known as MRFs, consisting of MyoD, myogenin, Myf5 and MRF4 (Myf6), are considered to be the master regulators of skeletal myogenesis [4]. It has been reported that the peak expression of Akirin1 is observed considerably earlier than MyoD during myoblast differentiation, indicating that Akirin1 functions upstream of MyoD. Over-expression of Akirin1 in C2C12 cells induces the withdrawal from the cell cycle and accelerates the expression of differentiation markers MyoD, Myogenin and MHC [14,19]. Conversely, knock-down of Akirin1 by RNAi down-regulates the expression of MyoD and myogenin in C2C12 myoblasts [14]. More recently, Nowak *et al.* identified Akirin as a factor that facilitates an interaction between Twist and the Brahma-containing (BRM) chromatin remodeling complex to promote gene expression [27]. Whether Akirin interacts with Twist to positively regulate the expression of MRFs will be necessary in future investigations.

Akirin2, a member of Akirin family, also plays an important role in skeletal myogenesis and affects the myogenic differentiation in birds. In the absence of Akirin1, the function of Akirin in skeletal muscle growth may be fulfilled by Akirin2 in birds [7]. It will be necessary to determine whether Akirin2 could regulate skeletal myogenesis in other animals in future investigations. Together, these findings indicate that Akirin plays an important role in myogenic differentiation, and it promotes skeletal myogenesis through up-regulation of MRFs expression.

### 4.3. Akirin Decreases the Expression of CD34 and Sca-1

During differentiation, a large subpopulation of myoblasts remains quiescent but is capable of self-renewal and differentiation. This subpopulation is called reserve cells and expresses stem cell antigen-1 (Sca-1) and CD34, the markers of satellite cells [28,29]. It has been shown that Sca-1 is associated with maintaining cell in non-differentiation and slow proliferation state, while CD34 is associated with keeping reserve cells out of fusion [30]. There is considerable evidence that Sca-1 and CD34 play an important role in myogenic differentiation [30,31]. In C2C12 cells, the expression of Sca-1 and CD34 were greater in the reserve cell population than in the myotube population [16]. There is a body of evidence to suggest that Akirin1 over-expression may cause the enhanced fusion phenotype and myoblast hypertrophy [16]. It may be explained that Akirin1 over-expression in C2C12 cells significantly reduced the expression of CD34 and Sca-1 [14,18]. Moreover, molecular and immunohistological analysis indicated that low levels of *Akirin1* are associated with quiescent satellite cells, while higher levels of Akirin1 are detected in activated proliferating satellite cells [16]. The results indicate that Akirin might be associated with satellite cells activation.

Based on the above, we can conclude that the Akirin is a downstream target gene of myostatin and a critical promyogenic factor, regulating the expression of myogenic differentiation related genes. Additionally, it also regulates key steps of muscle regeneration such as chemotaxis of inflammatory cells, satellite cells activation and migration. However, the precise function and molecular mechanisms of Akirin in skeletal myogenensis remains unclear. In addition, Akirin regulated skeletal myogenesis is mainly concentrated on *Akirin1* gene, so the relationship between *Akirin2* gene and skeletal myogenesis needs to be characterized. Therefore, further studies are required to elucidate the function and molecular basis of Akirin in the control of skeletal muscle development of livestock and poultry.

## 5. Akirin Signaling Pathway

The signaling pathway for Akirin remains large unknown. At present, there is growing evidence that Akirin1 can stimulate skeletal muscle cell differentiation and enhance the myotube formation through IGF-II-PI3K-Akt signaling pathway [14]. As a differentiation inducing factor, IGF-II has been shown to enhance the differentiation of myoblasts and the formation of myotubes [32]. It has been reported that over-expression of Akirin1 in C2C12 myoblasts increases the secretion of IGF-II during the myogenic differentiation, and the phosphorylated Akt was also elevated by Akirin1 over-expression [14]. When blocking PI3K pathway by the PI3K specific inhibitor LY294002, the *Akirin1* promoter activity is inhibited [14]. Taken together, these results provide evidence that Akirin1 regulates skeletal myogenesis via IGF-II-PI3K-Akt signaling pathway. Further research is required to investigate whether other pathways are involved in the Akirin-mediated skeletal myogenesis.

## 6. Conclusions and Future Directions

Skeletal myogenesis has been widespread concern, which is mainly regulated by muscle transcription factors such as MRFs and MEF2. Akirin is a recently discovered nuclear factor that plays an important role in skeletal myogenesis. However, its precise mechanisms are still lacking. Therefore, intensive studies of the molecular mechanism on how Akirin exerts its role in skeletal myogenesis will be critical not only for understanding the control of skeletal myogenesis but also for improving animal meat yield in livestock production.
